# Rural adolescent attitudes and use of helmets while snowmobiling

**DOI:** 10.1186/s40621-025-00648-z

**Published:** 2026-01-27

**Authors:** Brianna J. Iverson, Devin E. Spolsdoff, Pam J. Hoogerwerf, Brenda Vergara, Charles A. Jennissen

**Affiliations:** 1 Roy J. and Lucille A. Carver College of Medicine, University of Iowa, Iowa City, IA USA; 2 Department of Emergency Medicine, Roy J. and Lucille A. Carver College of Medicine, University of Iowa, Iowa City, IA USA; 3 Injury Prevention and Community Outreach, University of Iowa Stead Family Children’s Hospital, University of Iowa, Iowa City, IA USA; 4 Stead Family Department of Pediatrics, Roy J. and Lucille A. Carver College of Medicine, University of Iowa, Iowa City, IA USA; 5 Department of Emergency Medicine, University of Iowa Hospitals and Clinics, 200 Hawkins Dr, Iowa City, IA 52242 USA

**Keywords:** Adolescent, Helmet, Farm, Laws, Rural, Snowmobile, Teenager

## Abstract

**Background:**

Helmet-preventable head injuries are a common cause of snowmobiling-related injury. Our objective was to determine the demographics, helmet use, and attitudes regarding snowmobile helmet use among rural adolescents.

**Methods:**

An anonymous survey was administered to a convenience sample of adolescents (ages 13–18) at the 2022 Iowa FFA Leadership Conference. Frequency and comparative analyses were performed.

**Results:**

Of the 1,331 respondents, 58% were female and 96% non-Hispanic White. One-half lived on farms, 21% lived in the country/not on a farm, and 28% lived in town. One-quarter (26%) lived in households owning a snowmobile, with higher ownership among farm residents (31%) compared to those in the country/not on a farm (23%) or in town (19%), *p* < 0.001. Over one-third of participants had ridden in the past year. Riding was more common amongst males, Caucasians, farm residents, and those from households owning snowmobiles (all *p* ≤ 0.01). Frequent riding (at least weekly) was higher among older teens and snowmobile-owning households (*p* = 0.025 and *p* < 0.001, respectively). Helmet use was: 67% always/mostly, 11% sometimes, and 21% rarely/never. The importance of snowmobile helmet use (from 1 to 10) was highly rated (median, 9; mean, 8.2). Relative to their peers, females (*p* = 0.018), those owning snowmobiles (*p* < 0.001), and frequent riders (*p* < 0.01) had greater proportions wearing helmets always/most of the time, and rated the importance of helmet use more highly. 59% supported snowmobile helmet laws.

**Conclusion:**

While most rural adolescents value snowmobile helmet use and support legislation, nearly half report inconsistent use. Importantly, our study identified demographic groups for targeted interventions.

## Background

Snowmobiling is a major recreational activity in North America with about 1.2 million snowmobiles registered in the United States (U.S.) and 555,000 in Canada [1]. Within Iowa, there were over 21,000 snowmobiles registered as of 2024, and snowmobiling is used as a method of transportation in many rural areas with snow cover during the winter season [1]. Snowmobile-related tourism generates over $26 billion in annual spending and provides over 100,000 jobs in the U.S. The majority of this spending occurs in rural areas [2].

The continued interest and growth of the sport has resulted in a corresponding increase in snowmobile-related deaths and injuries. An estimated 200 fatalities and an additional 14,000 injuries requiring emergency department (ED) visits occur annually in North America due to snowmobiles [3]. Many fatalities associated with snowmobiles are due to head, neck or facial injury. Nearly a third of individuals who experience a snowmobile injury require intensive care unit (ICU) admission, and head trauma is the leading cause of snowmobile-related deaths in children under the age of seventeen [3–5].

Snowmobile helmets are effective in reducing and preventing head injury [6–8]. For example, Nayci et al. conducted a retrospective analysis of snowmobile-related injuries among children and adolescents treated at a Level 1 pediatric trauma center over a 10-year period. Of 107 patients, 69% sustained head injuries, and only 54% were documented as wearing helmets at the time of the crash [7]. Helmeted riders had significantly lower Injury Severity Scores (ISS) and fewer ICU admissions than unhelmeted riders (*p* < 0.05) [7]. This and other studies not only emphasize the protective effect of helmets but also highlight lack of consistent use, including among youth populations [6–10]. However, these studies are retrospective and focus on injured patients rather than assessing general patterns of helmet use or attitudes among the broader adolescent population.

As compared to other motorized vehicles like ATVs, snowmobile-related injuries and helmet use are rarely studied. Most existing data and analyses surrounding snowmobiles are limited and out of date [5, 6, 10–15]. To our knowledge, no previous studies have examined U.S. adolescents’ attitudes towards snowmobile helmet laws and the importance of wearing a helmet on snowmobiles, specifically among rural adolescents.

Previous studies have identified several factors associated with helmet use in motorized recreational activities. Demographic variables such as age and sex have been consistently linked to helmet-wearing behaviors, with younger males demonstrating lower rates of compliance [8, 12, 14]. Frequency of riding and perceived risk have also been shown to influence helmet use – individuals who ride more frequently or who perceive snowmobiling as less risky are less likely to wear helmets consistently [15–17]. In addition, policy and enforcement play an important role. In the United States, snowmobile helmet laws vary by state, and many lack universal helmet requirements. Evidence from Canada and several northern European countries suggests that mandatory helmet legislation combined with public education campaigns leads to substantially higher helmet use and reductions in head injury incidence [18–20]. These findings helped guide us in the selection of our study variables.

To reduce snowmobile-related injuries and fatalities, we need to better understand current patterns of helmet use and rider’s attitudes toward wearing head protection. This will allow the development of more effective strategies to increase use. Our primary study objective was to determine the frequency of snowmobile helmet use in rural adolescents. Additionally, we wanted to investigate their beliefs about the importance of helmets, their attitudes towards helmet laws, and to identify demographic factors associated with helmet usage while snowmobiling.

## Methods

### Study population

A cross-sectional survey design was utilized with a convenience sample of 2022 Iowa FFA (formerly known as Future Farmers of America) Leadership Conference attendees in Ames, Iowa. FFA is a school-affiliated intramural organization that provides young people across all 50 states and Puerto Rico with opportunities for growth in agricultural science, business, and technology. FFA members who passed by or visited the University of Iowa Stead Family Children’s Hospital (SFCH) injury prevention booth were invited by volunteers to complete an anonymous survey. Surveys were self-administered, either on paper or via a digital Qualtrics form, and paper surveys were subsequently checked for completeness by the injury prevention booth staff. Upon finishing the survey, participants could play a Plinko game to win a small prize as an incentive.

### Survey

The survey was created by the University of Iowa SFCH Injury Prevention and Community Outreach program in partnership with community stakeholders, with the goal of collecting data to support rural adolescent helmet safety initiatives. The survey covered helmet use on a variety of modalities including bicycles, ATVs, motorcycles, dirt bikes, horses and snowmobiles. However, this manuscript will specifically address findings related to snowmobile helmet use. To ensure clarity and validity, the survey was piloted among adolescents aged 13 to 21 years, allowing for feedback on potential areas needing revision. Collected demographic information included participants’ age (years old), sex/gender (male, female, nonbinary, other), living situation (farm, country but not on a farm, town), and race/ethnicity (White/Caucasian, Black/African American, Latino/Latinx, Asian, or other).

Participants were asked whether their family owned a snowmobile (yes, no) and how often they went snowmobiling (not in the past year, a few times a year or less, weekly, monthly, daily). Because snowmobiling is a seasonal activity, responses regarding frequency (e.g., “daily” or “weekly”) referred to use during the active snowmobiling season rather than year-round behavior. For those who had ridden a snowmobile within the past year, follow-up questions assessed the frequency of helmet use during rides (always, most of the time, sometimes, rarely, or never). All respondents also rated the perceived importance of helmet use while snowmobiling on a scale from 1 to 10, where 1 indicated “not at all important,” 5 signified “moderately important,” and 10 represented “extremely important.” Additionally, participants were asked if they believed there should be a law requiring a helmet when riding a snowmobile (yes, no).

### Data analysis

The research team received all completed surveys for analysis. The study was determined to be exempt by the Institutional Review Board, as it involved the use of pre-existing, anonymous data. Paper surveys were entered into Qualtrics and combined with digital responses collected via mobile devices during the conference. The compiled dataset was exported and analyzed in the software program, R (https://www.r-project.org/). While all conference participants were invited to complete the survey, data analysis was limited to individuals aged 13 to 18 years. Missing values were excluded from analyses.

Analytical methods included descriptive statistics (frequencies), bivariate analyses (chi-square tests and Fisher’s exact tests), and multivariable logistic regression modeling. Depending on the number of groups compared, the Wilcoxon rank sum test or Kruskal-Wallis test was used to determine difference in medians. Due to a lack of racial and ethnic diversity in the sample, the race/ethnicity variable was dichotomized into “non-Hispanic White” and “other” categories. While this approach resulted in notable heterogeneity within the “other” group, it permitted inclusion of the variable in multivariable models. Statistical significance was defined as a two-sided p-value of less than 0.05.

## Results

### Study demographics

1,331 adolescents participated with 58% being females and 56% being 16–18 years of age. See Table 1 for demographics of the survey respondents. One-half lived on a farm, 21% lived in the country but not on a farm, and 28% resided in town. The vast majority of participants (96%) identified as non-Hispanic White.

**Table 1:** Demographics of survey respondents at the 2022 Iowa FFA Leadership Conference

	*n* (Col %)^a^
Group N	1,331
Sex	
Male Female Nonbinary	543 (41%) 770 (58%) 16 (1%)
Age	
13 years 14 years 15 years 16 years 17 years 18 years	66 (5%) 171 (13%) 337 (26%) 300 (23%) 272 (21%) 166 (13%)
Residence	
Farm Country/not farm Town	670 (50%) 285 (21%) 376 (28%)
Race/ethnicity	
non-Hispanic White Black/African American Asian Latino/Latinx Other	1,320 (96%) 17 (1%) 8 (0.5%) 35 (3%) 13 (1%)
Riding frequency	
Daily	33 (3%)
Weekly	52 (4%)
Monthly	66 (5%)
Few times a year or less	298 (23%)
Have not ridden	828 (65%)
Helmet use	
Always	240 (53%)
Mostly	65 (14%)
Sometimes	51 (11%)
Rarely	27 (6%)
Never	66 (15%)

^a^The sum of n may not equal the total Group N due to missing values, and percentages may not add up to 100% due to rounding

### Snowmobile ownership

Over one-fourth (26%) of respondents lived in a household that owned a snowmobile. See Table 2 for bivariate and multivariable logistic regression analysis regarding snowmobile ownership. A higher proportion of those living on farms owned a snowmobile (31%) than those from the country but not on a farm (23%) or from towns (19%), *p* < 0.001.

**Table 2:** Bivariate and multivariable logistic regression analyses regarding whether the household owned a snowmobile among survey respondents at the 2022 Iowa FFA Leadership Conference

	Bivariate analysis			Logistic regression analysis^a^	
	Yes *n* (Row %)^b^	No *n* (Row %)^b^	*p*-value	aOR	95% CI
Group N	331 (26%)	963 (74%)			
Sex			0.364		
Male	143 (27%)	383 (73%)		1.15	(0.89–1.49)
Female	186 (25%)	565 (75%)		1.0 (ref)	
Age			0.204		
16–18 years 13–15 years	194 (27%) 132 (24%)	525 (73%) 425 (76%)		1.16 1.0 (ref)	(0.89–1.50)
Residence			**< 0.001**		
Farm Country/not farm	199 (31%) 63 (23%)	451 (69%) 213 (77%)		**1.83** 1.19	**(1.33–2.52)** (0.80–1.76)
Town	69 (19%)	299 (81%)		1.0 (ref)	
Race/ethnicities			0.071		
non-Hispanic White Other races/ethnicities	320 (26%) 11 (16%)	903 (74%) 59 (84%)		1.52 1.0 (ref)	(0.78–2.98)

aOR, adjusted odds ratio; CI, confidence interval

^a^The total number of cases used in the logistic regression model was 1260

^b^The sum of n for a variable may not equal the total Group N due to missing values

Values of significant difference are bolded

### Snowmobile use

More than one-third (35%) of respondents reported riding a snowmobile in the past year (23% a few times a year or less, 5% monthly, 4% weekly and 3% daily). See Table 1. Males, those living on farms, non-Hispanic Whites and those whose families owned snowmobiles all had greater percentages that had ridden a snowmobile in the past year as compared to their peers, all *p* < 0.01. See Table 3 for bivariate and multivariable logistic regression analysis regarding whether an adolescent had ridden a snowmobile or not in the past year. However, logistic regression analysis controlling for other variables found no differences by residence location and older teens had one-third less odds of having ridden a snowmobile in the last year as compared to younger teens. Snowmobile owners had 52 times greater odds of having ridden snowmobiles in the past year as compared to non-owners.

**Table 3:** Bivariate and multivariable logistic regression analyses regarding whether they had ridden a snowmobile in the past year among survey respondents at the 2022 Iowa FFA Leadership Conference

	Bivariate analysis				Logistic regression analysis^a^		
	Ridden *n* (Row %)^b^	Never ridden *n* (Row %)^b^	*p*-value		aOR	95% CI	
Group N	449 (35%)	828 (65%)					
Sex			**0.001**				
Male	211 (39%)	332 (61%)			**1.95**	**(1.40–2.70**)	
Female	231 (30%)	539 (70%)			1.0 (ref)		
Age			0.402				
16–18 years 13–15 years	241 (33%) 201 (35%)	497 (67%) 373 (65%)			**0.66** 1.0 (ref)	(**0.48–0.92**)	
Residence			**0.002**				
Farm Country/not farm	256 (38%) 83 (29%)	414 (62%) 202 (71%)			0.94 0.64	(0.65–1.37) (0.40–1.04)	
Town	110 (29%)	266 (71%)			1.0 (ref)		
Race/ethnicity			**0.010**				
non-Hispanic White Other races/ethnicities	435 (35%) 14 (19%)	822 (65%) 59 (81%)			**2.95** 1.0 (ref)	**(1.20–7.27)**	
Snowmobile ownership			**< 0.001**				
Yes	296 (89%)	35 (11%)			**52.67**	**(34.64–80.08)**	
No	153 (16%)	810 (84%)			1.0 (ref)		

aOR, adjusted odds ratio; CI, confidence interval

^a^The total number of cases used in the logistic regression model was 1243.

^b^The sum of n for a variable may not equal the total Group N due to missing values.

Values of significant difference are bolded

Of those who had ridden snowmobiles in the past year, 81% rode monthly or less and 19% rode at least weekly. See Table 4 for bivariate and multivariable logistic regression analysis regarding riding frequency. Older teens and snowmobile owners had higher proportions riding at least weekly as compared to their peers, both *p* < 0.03. Respondents whose families owned snowmobiles had nearly 11 times greater odds of riding at least weekly as compared to non-owners.

**Table 4:** Bivariate and multivariable logistic regression analyses regarding frequency of riding a snowmobile among survey respondents at the 2022 Iowa FFA Leadership Conference

	Bivariate analysis				Logistic regression analysis^a^	
	Few times/monthly *n* (Row %)^b^	Weekly/daily *n* (Row %)^b^	*p*-value		aOR	95% CI
Group N	364 (81%)	85 (19%)				
Sex						
Male	161 (76%)	50 (24%)	0.090		**2.28**	**(1.35–3.86)**
Female	167 (84%)	33 (16%)			1.0 (ref)	
Age			**0.025**			
16–18 years 13–15 years	154 (75%) 170 (85%)	51 (25%) 31 (15%)			1.23 1.0 (ref)	(0.73–2.09)
Residence			0.081			
Farm Country/not farm	198 (77%) 66 (88%)	58 (23%) 9 (12%)			1.28 0.50	(0.67–2.46) (0.20–1.25)
Town	92 (84%)	18 (16%)			1.0 (ref)	
Race/ethnicity			0.254			
non-Hispanic White Other races/ethnicities	354 (81%) 8 (67%)	81 (19%) 4 (33%)			0.83 1.0 (ref)	(0.20–3.47)
Snowmobile ownership			**< 0.001**			
Yes	219 (74%)	77 (26%)			**10.71**	**(4.17–27.51)**
No	145 (95%)	8 (5%)			1.0 (ref)	

aOR, adjusted odds ratio; CI, confidence interval

^a^The total number of cases used in the logistic regression model was 460, only includes those that had ridden a snowmobile

^b^The sum of n for a variable may not equal the total Group N due to missing values

Values of significant difference are bolded

### Helmet use and comparison

Over half (53%) of snowmobile riders stated they always wore a helmet with another 14% reporting mostly wearing a helmet. See Table 1. About one-third reported wearing helmets sometimes or less. Females had higher proportions that always/mostly wore a helmet as compared to males, *p* = 0.018. Those whose families owned a snowmobile had higher percentages always/mostly wearing a helmet as compared to non-owners (78% vs. 49%), *p* < 0.001. Frequent riders (weekly/daily) had higher proportions always/mostly wearing a helmet than less frequent riders (84% vs. 64%), *p* = 0.001. See Table 5 for bivariate and multivariable logistic regression analysis regarding whether a helmet is worn for those who had ridden a snowmobile in the past year. Participants whose families owned a snowmobile had over 3 times greater odds of always/mostly wearing helmets versus non-owners.

**Table 5:** Bivariate and multivariable logistic regression analyses regarding whether a helmet is worn for those who had ridden a snowmobile in the past year among survey respondents at the 2022 Iowa FFA Leadership Conference

	Bivariate analysis				Logistic regression analysis^a^	
	Always/mostly *n* (Row %)^b^	Never/rarely/sometimes *n* (Row %)^b^	*p*-value		aOR	95% CI
Group N	305 (68%)	144 (32%)				
Sex			**0.018**			
Male	133 (63%)	78 (37%)			**0.60**	**(0.39–0.93)**
Female	170 (74%)	60 (26%)			1.0 (ref)	
Age			0.612			
16–18 years 13–15 years	168 (70%) 134 (67%)	73 (30%) 66 (33%)			0.93 1.0 (ref)	(0.60–1.44)
Residence			0.588			
Farm Country/not farm	178 (70%) 53 (64%)	77 (30%) 30 (36%)			0.86 0.80	(0.51–1.47) (0.41–1.57)
Town	74 (67%)	36 (33%)			1.0 (ref)	
Race/ethnicity			0.075			
non-Hispanic White Other races/ethnicities	299 (69%) 6 (43%)	135 (31%) 8 (57%)			**4.14** 1.0 (ref)	**(1.22–14.09)**
Snowmobile ownership			**< 0.001**			
Yes	230 (78%)	66 (22%)			**3.13**	**(1.98–4.96)**
No	75 (49%)	77 (51%)			1.0 (ref)	
Riding frequency			**0.001**			
Monthly/few times	234 (64%)	129 (36%)			1.0 (ref)	
Daily/weekly	71 (84%)	14 (16%)			**2.71**	** (1.31–5.58)**

aOR, adjusted odds ratio; CI, confidence interval

^a^The total number of cases used in the logistic regression model was 435, only includes those that had ridden a snowmobile

^b^The sum of n for a variable may not equal the total Group N due to missing values

Values of significant difference are bolded

### Helmet law

The majority (59%) of study adolescents believed there should be a law requiring helmet use on snowmobiles. See Table 6 for bivariate and multivariable logistic regression analysis regarding whether respondents supported a helmet law. Females and those whose families owned snowmobiles had higher percentages that supported a snowmobile helmet law than their peers, both *p* < 0.02.

**Table 6:** Bivariate and multivariable logistic regression analyses regarding whether they supported a snowmobile helmet law among survey respondents at the 2022 Iowa FFA Leadership Conference

	Bivariate analysis				Logistic regression analysis^a^	
	Law *n* (Row %)^b^	No law *n* (Row %)^b^	*p*-value		aOR	95% CI
Group N	757 (59%)	519 (41%)				
Sex			**< 0.001**			
Male	241 (46%)	282 (54%)			**0.40**	**(0.32–0.51)**
Female	505 (69%)	231 (31%)			1.0 (ref)	
Age			0.130			
16–18 years	407 (58%)	299 (42%)			0.83	(0.65–1.05)
13–15 years	343 (62%)	210 (38%)			1.0 (ref)	
Residence			0.109			
Farm	368 (57%)	273 (43%)			**0.72**	**0.54–0.96**
Country/not farm	157 (58%)	115 (42%)			0.80	(0.57–1.13)
Town	232 (64%)	131 (36%)			1.0 (ref)	
Race/ethnicity			0.534			
non-Hispanic White	719 (60%)	487 (40%)			1.36	(0.80–2.31)
Other races/ethnicities	38 (55%)	31 (45%)			1.0 (ref)	
Snowmobile ownership			**0.018**			
Yes	214 (65%)	115 (35%)			**2.07**	**(1.42–3.01)**
No	543 (57%)	403 (43%)			1.0 (ref)	
Ridden in past year			0.388			
Yes	257 (58%)	189 (42%)			**0.63**	**(0.45–0.89)**
No	495 (60%)	326 (40%)			1.0 (ref)	
Riding frequency^c^						
Monthly/few times	204 (57%)	157 (43%)	0.390		---	
Daily/weekly	53 (62%)	32 (38%)			---	

aOR, adjusted odds ratio; CI, confidence interval

^a^ The total number of cases used in the logistic regression model was 1234

^b^ The sum of n for a variable may not equal the total Group N due to missing values

^c^ Not used in the logistic regression analysis

Values of significant difference are bolded

### Helmet importance

Overall, the importance of helmet use (1–10, 10 “extremely important”) while riding snowmobiles had a mean of 8.2 and a median of 9. See Table 7 for variable comparisons in the median importance ascribed to wearing a helmet while riding a snowmobile. Females, those in households that owned snowmobiles, and those supporting a snowmobile law all rated the importance of wearing a helmet more highly than their peers, all *p* < 0.002. The greater the use of snowmobile helmets, the higher the median helmet importance rating with those always wearing a helmet rating the importance at 10 and those never wearing a helmet rating it at 4, *p* < 0.001.

**Table 7:** Variable comparisons in the median importance ascribed to wearing a helmet while riding a snowmobile among survey respondents at the 2022 Iowa FFA Leadership Conference

	Median importance (1–10)^a^	*p*-value
Overall	9	
Sex		**< 0.001**
Male	8	
Female	10	
Age		0.643
16–18 years 13–15 years	9 9	
Residence		0.160
Farm Country/not farm	9 10	
Town	9	
Race/ethnicity		0.709
non-Hispanic White	9	
Other races/ethnicities	10	
Snowmobile ownership		**0.001**
Yes	10	
No	9	
Ridden in past year		0.106
Yes	9	
No	10	
Riding frequency		0.157
Monthly/few times	9	
Daily/weekly	10	
Helmet use		**< 0.001**
Never Sometimes/rarely	4 7	
Most of the time	8	
Always	10	
Helmet law opinion		**< 0.001**
Support Against	10 7	

^a^Median importance was 1–10, with 1 being “not at all important” and 10 being “extremely important”

Values of significant difference are bolded

## Discussion

This study of rural Iowa adolescents found that more than a quarter of their families owned a snowmobile and that over a third (35%) had ridden a snowmobile in the past year. Respondents that lived on farms had higher proportions owning and riding snowmobiles. Males, non-Hispanic Whites and those owning snowmobiles also had higher percentages having ridden a snowmobile in the past year. Helmet use on snowmobiles was relatively high with over two-thirds stating they always or mostly wore a helmet, and the median importance ascribed to wearing a helmet while snowmobiling was 9, where 10 was extremely important. Our results showed a direct relationship between helmet use and the perceived importance of wearing a helmet. Females, frequent riders, and snowmobile owners all had higher proportions that always/mostly wore helmets and viewed helmet use as highly important. Moreover, three-fifths (59%) of respondents supported a law mandating helmet use when riding snowmobiles.

### Helmet use

Slightly over half of study participants reported they always wore a helmet when snowmobiling. This proportion is similar to other studies that reported snowmobile helmet use between 50 and 65% [7, 10, 11, 16–18]. In this survey of Iowa FFA members, we also asked about helmet use on other motorized vehicles. We found that the proportion always or mostly wearing a helmet on snowmobiles (59%) was the highest when compared to ATVs (21%), dirt bikes (51%), and motorcycles (58%) [16]. Another study examined over 750 injuries associated with snowmobiles, ATVs, and dirt bikes seen at the Mayo Clinic in Minnesota. Consistent with our findings, it reported low rates of helmet use among ATV riders, while helmet use among snowmobile riders was notably higher [19].

One factor affecting higher helmet use may be the colder temperatures outside when adolescents are riding snowmobiles. Staying warm is important for having fun while snowmobiling so “bundling up” with a coat, gloves, hat, and a helmet can be comforting. Other winter sports such as skiing and snowboarding also have greater helmet use rates similar to snowmobilers [12, 13, 18, 20–24]. However, in a survey investigating helmet use in children who skied or snowboarded, nearly 90% reported that they wore helmets for safety reasons [25], and another study found that the protective benefits of helmets was the primary reason adolescents wore them [26].

We found that the median importance ascribed to wearing a helmet while snowmobiling was 9, which is quite high. A previous published analysis of the same survey regarding ATV, dirt bike and motorcycle data showed a median importance of only 6 for ATVs [13]. Moreover, adolescents whose families owned snowmobiles ascribed greater importance to wearing helmets as compared to non-owners. This is in contrast to ATVs, dirt bikes and motorcycles where non-owners rated the importance of wearing helmets higher than owners [13]. The higher importance rating and greater use of helmets while snowmobiling, as compared to activities like riding ATVs, suggests a better safety culture surrounding this sport.

### Safety training

The state of Iowa requires adolescents aged 12–17 years to complete a snowmobile safety certification course if planning to snowmobile on public land or ice [27]. Such education may have a positive impact on the safety culture surrounding helmet use while snowmobiling. In Nova Scotia, helmet use increased to 90% after a social media and educational campaign that focused on helmet safety for skiers [18]. Other programs to educate riders include the Alberta Snowmobile Association’s Sled Smart program and the Snowmobile Trail Officer Patrol program [14, 28]. Although educational programs are likely beneficial, evidence supporting their effectiveness in preventing snowmobile-related injury is still lacking [11]. For example, a high rate of adolescent snowmobiler injuries persists despite the state of New York requiring operator training [8]. Further investigation is needed to assess how snowmobile safety programs might be improved. Our findings suggest that certain demographic groups—such as males and infrequent snowmobiler users—reported lower helmet use. These populations may represent key targets for intervention through community-based safety programs and regionally tailored education efforts.

### Helmet law

Although snowmobilers do report higher helmet use than riders of some other vehicles, our study still found that almost 50% of adolescents were not always wearing a helmet. Unhelmeted snowmobilers are more likely to die or experience severe morbidity from their injuries [3, 4, 7, 8, 15]. Laws requiring helmet use on snowmobiles may increase helmet use, especially if they are universal (involving all ages) and pertain to both public and private land [15]. We found that three-fifths (59%) of study adolescents supported a law requiring helmets while riding snowmobiles, and that those whose families owned a snowmobile had greater support for a law than non-owners. This was not true for ATVs, dirt bikes and motorcycles where owners expressed lower support for helmet laws than non-owners [16]. This suggests the public may be more amenable to passage of laws requiring helmets for snowmobiles than for some other motorized vehicles.

## Limitations

Our study involved a convenience sample of adolescent FFA members from a predominantly rural Midwestern state. As such, the results may not reflect the broader state population, particularly adolescents from urban areas. However, the majority of counties in the state were represented by participants. Findings may also not be generalizable to other regions of the United States. In particular, given the predominantly non-Hispanic White demographic, the findings may not extend to more racially or ethnically diverse populations. In addition, self-reported data may be subject to recall and social desirability biases. The use of anonymous, independently completed written surveys should have helped minimize the latter. Additionally, the study was performed on secondary data.

## Conclusions

Snowmobiling remains a popular rural recreational activity, yet it is associated with significant risk for head trauma, particularly in youth. While previous studies have examined snowmobile-related injuries, there remains a paucity of recent data on helmet use among adolescent riders [3–5, 7]. Our study fills a critical gap by characterizing helmet use patterns and attitudes in a large, rural youth cohort. Compared to similar research on helmet use for ATVs and dirt bikes, our findings suggest a more robust safety culture surrounding snowmobiling, with higher rates of consistent helmet use and stronger support for helmet legislation [16]. Still, nearly half of adolescent snowmobile riders reported inconsistent helmet use, highlighting an urgent need for targeted interventions. These findings underscore the role sports medicine professionals and public health advocates can play in injury prevention through education, policy support, and community engagement. Promoting helmet use through both legislation and culturally tailored outreach may reduce the burden of snowmobile-related head injuries in rural populations. Future research should assess the effectiveness of specific interventions, including safety training and helmet mandates, to inform best practices in youth sports injury prevention.

## Data Availability

Data and materials are available to other parties for research purposes after a data sharing agreement plan is agreed to and signed. Those interested should contact the corresponding author.

## References

[1] International Snowmobile Manufacturers Association. Snowmobiling fact book. 2022. Available at: http://www.snowmobile.org/docs/isma-snowmobilint-fact-book.pdf

[2] American Council of Snowmobile Associations. Facts and myths about snowmobiling on winter trails. 2022. Available at: https://www.snowmobilers.org/docs/acsa_facts_and_myths_book.pdf

[3] Pierz JJ. Snowmobile injuries in North America. Clin Orthop Relat Res. 2003;409:29–36. 10.1097/01.blo.0000057781.10364.c9 12671482

[4] Whiting P, et al. Orthopaedic injuries from snowmobile accidents: a multi-centre analysis of demographics, injury patterns, and outcomes. Eur J Orthop Surg Traumatol. 2019;29(8):1617–21. 31359179 10.1007/s00590-019-02514-3 PMC8048394

[5] Rice MR, Alvanos L, Kenney B. Snowmobile injuries and deaths in children: a review of national injury data and state legislation. Pediatrics. 2000;105(3 Pt 1):615–9. 10699118 10.1542/peds.105.3.615

[6] Skokan EG, Junkins EP, Kadish H. Serious winter sport injuries in children and adolescents requiring hospitalization. Am J Emerg Med. 2003;21(2):95–9. 12671807 10.1053/ajem.2003.50032

[7] Nayci A, et al. Snowmobile injuries in children and adolescents. Mayo Clin Proc. 2006;81(1):39–44. 16438477 10.4065/81.1.39

[8] Plog BA, et al. Neurologic injury in snowmobiling. Surg Neurol Int. 2014;5:87. 25024887 10.4103/2152-7806.134074 PMC4093746

[9] Milan M, et al. Helmet use and injury severity among pediatric skiers and snowboarders in Colorado. J Pediatr Surg. 2017;52(2):349–53. 27876383 10.1016/j.jpedsurg.2016.11.001

[10] Koskinen K. Snowmobile traumas in Finnish Lapland: injuries to head, face and neck. Possible effects of speed and the use of helmet. Arct Med Res. 1994;53(Suppl 3):5–7. 7710593

[11] Beilman GJ, et al. Risk factors and patterns of injury in snowmobile crashes. Wilderness Environ Med. 1999;10(4):226–32. 10628282 10.1580/1080-6032(1999)010[0226:rfapoi]2.3.co;2

[12] Lawrence L, Shaha S, Lillis K. Observational study of helmet use among children skiing and snowboarding. Pediatr Emerg Care. 2008;24(4):219–21. 18431220 10.1097/PEC.0b013e31816a9f0a

[13] Hackam D.J., Kreller M., Pearl R.H. Snow-related recreational injuries in children: assessment of morbidity and management strategies. J Pediatr Surg. 1999;34(1):65–8. discussion 69. 10022145 10.1016/s0022-3468(99)90230-0

[14] Hoey J. Snowmobile injuries. CMAJ. 2003;168(6):739. 12642432 PMC154924

[15] James EC, et al. Snowmobile trauma: an eleven-year experience. Am Surg. 1991;57(6):349–53. 2048843

[16] Jennissen CA, et al. Rural adolescent attitudes and use of helmets while riding ATVs, motorcycles and dirt bikes. Inj Epidemiol. 2024;11(Suppl 1):44. 39237989 10.1186/s40621-024-00532-2 PMC11375824

[17] Srinivas S, et al. Pediatric snow sport injuries differ by age. J Pediatr Surg. 2021;56(3):520–5. 32653163 10.1016/j.jpedsurg.2020.05.034

[18] Fenerty L, et al. Achieving all-age helmet use compliance for snow sports: strategic use of education, legislation and enforcement. Inj Prev. 2016;22(3):176–80. 26658338 10.1136/injuryprev-2015-041699

[19] Shannon SF, et al. Pediatric orthopaedic trauma and associated injuries of snowmobile, ATV, and dirtbike accidents: a 19-year experience at a level 1 pediatric trauma center. J Pediatr Orthop. 2018;38(8):403–9. 27442216 10.1097/BPO.0000000000000838

[20] Sadeghian H, et al. Factors influencing helmet use, head injury, and hospitalization among children involved in skateboarding and snowboarding accidents. Perm J. 2017;21:16–161. 28406787 10.7812/TPP/16-161 PMC5391782

[21] Sulheim S, et al. Helmet use and risk of head injuries in alpine skiers and snowboarders: changes after an interval of one decade. Br J Sports Med. 2017;51(1):44–50. 27531522 10.1136/bjsports-2015-095798

[22] Lee LK, et al. Helmet use in preventing head injuries in bicycling, snow sports, and other recreational activities and sports. Pediatrics. 2022. 10.1542/peds.2022-058877. 35965276 10.1542/peds.2022-058878

[23] Sulheim S, et al. Helmet use and risk of head injuries in alpine skiers and snowboarders. JAMA. 2006;295(8):919–24. 16493105 10.1001/jama.295.8.919

[24] Hagel BE, et al. Effectiveness of helmets in skiers and snowboarders: case-control and case crossover study. BMJ. 2005;330(7486):281. 15632094 10.1136/bmj.38314.480035.7C PMC548175

[25] Provance AJ, Engelman GH, Carry PM. Implications of parental influence on child/adolescent helmet use in snow sports. Clin J Sport Med. 2012;22(3):240–3. 22270869 10.1097/JSM.0b013e3182410335

[26] Fenerty L, et al. Helmets for skiing and snowboarding: who is using them and why. J Trauma Acute Care Surg. 2013;74(3):895–900. 23425754 10.1097/TA.0b013e31827e19ca

[27] Iowa Department of Natural Resources. Iowa snowmobile reference guide. 2025. Available at: https://www.iowadnr.gov/media/1578/download?inline

[28] Rowe BH, et al. The effect of a community-based police surveillance program on snowmobile injuries and deaths. Can J Public Health. 1998;89(1):57–61. 9524393 10.1007/BF03405797 PMC6990216

